# Optimizing the use of temporal artery biopsy: a retrospective study

**DOI:** 10.1186/s40463-022-00605-6

**Published:** 2023-01-26

**Authors:** Etienne Villeneuve, Jean-Michel Lacroix, Simon Brisebois

**Affiliations:** 1 Division of Otolaryngology, Head and Neck Surgery, Université de Sherbrooke, Sherbrooke, Canada; 2 Université de Sherbrooke, Sherbrooke, Canada

**Keywords:** Temporal artery biopsy, Giant cell arteritis, Temporal arteritis, Positivity rate, Jaw claudication

## Abstract

**Background:**

Giant cell arteritis is an inflammatory disease of the large- and medium-sized vessels. It is the most common primary vasculitis, with lifetime incidences of 0.5% and 1% in men and women, respectively. Its diagnosis is based upon clinical criteria, which may include temporal artery biopsy. Expected positivity rates of temporal artery biopsies and patient selection remain controversial topics in the literature.

**Methods:**

A cross-sectional retrospective study of 127 patients referred for temporal artery biopsy with a diagnosis of suspected giant cell arteritis between January 2014 and December 2018 was performed. The primary outcome was the positivity rate. The relationships between positivity rates, symptoms, clinical suspicion, biopsy delay, biopsy length and corticosteroid treatment were also studied.

**Results:**

A positivity rate of 23.7% (16.6–32.6%) was shown, along with a significant association between jaw claudication and specimen positivity (odds ratio 8.1, *p* < 0.05). Moreover, there were significant associations between a high initial clinical suspicion of disease and specimen positivity (*p* < 0.05), as well as a high initial clinical suspicion of disease and pursuit of corticosteroid treatment following biopsy results, regardless of positivity (*p* < 0.05). The duration of corticosteroid treatment prior to biopsy was not associated with a change in positivity rate.

**Conclusions:**

The positivity rate of temporal artery biopsy was 23.7%. Treatment of patients with negative temporal artery biopsy was associated with maintenance of corticosteroid treatment when the initial clinical suspicion of arteritis was high. Therefore, temporal artery biopsy may not be necessary for patients with a high initial clinical suspicion of giant cell arteritis.

**Graphical Abstract:**

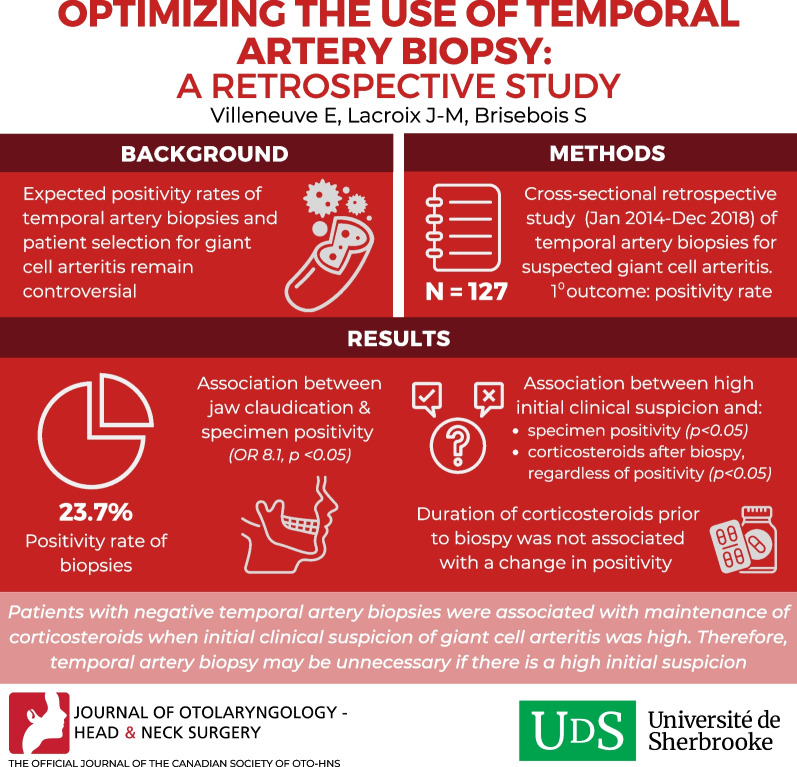

## Background

Giant cell arteritis is an inflammatory disease of the large- and medium-sized vessels, and it is the most common primary vasculitis. The lifetime incidences of developing the disease are 1% for women and 0.5% for men [1].

Symptoms associated with this clinical entity are nonspecific and may range from intense headaches, fevers, and jaw claudication to a permanent loss of vision, depending on the arteries affected. The risk of temporary or permanent blindness is estimated to be approximately 15–20% [2]. If treatment is started early, this risk can drop below 1% [3]. Loss of vision is usually permanent [4]. The main treatment consists of high-dose corticosteroids, such as prednisone, taken daily [5].

Diagnosis is based upon clinical criteria, which include temporal artery biopsy. In 1990, The American College of Rheumatology (ACR) established the following criteria for diagnosing giant cell arteritis [6].

1. Age at disease onset > or = to 50
2. New headache (new onset or new type of localized pain in the head)
3. Temporal artery abnormalities (tenderness at palpation or decreased pulsation, unrelated to arteriosclerosis of the cervical arteries)
4. Elevated erythrocyte sedimentation rate (> or = to 50 mm/hour by the Westergren method)
5. Abnormal artery biopsy (biopsy specimen with artery showing vasculitis characterized by a predominance of mononuclear cell infiltration or granulomatous inflammation, usually with multinucleated giant cells)

For classification purposes, diagnosis is confirmed for any patient presenting with three of the above criteria (93.5% sensitivity and 91.2% specificity). Therefore, a temporal artery biopsy should only be used if any two of the criteria are positive. However, these criteria have mostly been used for research purposes and vasculitis differentiation [7]. In a clinical context, temporal artery biopsies are frequently performed. This might also be influenced by prescription habits and local accessibility to the procedure. The specificity of temporal artery biopsy is 100%, which makes it a diagnostic gold standard. Unfortunately, false-negative results occur in 7% of patients [8], with certain studies showing higher rates of missed diagnoses. Excluding patients treated with steroids, the estimated sensitivity ranges from 77 to 87% [9, 10]. This is probably secondary to skip lesions, one of the main features of the disease [11].

Positivity rates of temporal artery biopsies are a topic of significant interest in the literature. Therefore, the main objective of the present study was to document temporal artery biopsy yields, aiming to establish a baseline for future studies. The researchers also wanted to explore the characteristics of patients requiring these biopsies and how these patients might be better selected for the procedure.

## Methods

Our study included all patients who had undergone a temporal artery biopsy in our center for a suspected diagnosis of giant cell arteritis over a five-year period (January 2014 to December 2018). The charts of 134 patients whose specimens were classified as “temporal arteries” in the pathologic reports were reviewed. Of these, seven were not included in the study because surgery had been performed for other purposes. Most of these were patients treated for cutaneous neoplasia in which resection included the superficial temporal artery. Otherwise, there were no exclusion criteria. A cross-sectional retrospective study involving these patients was performed, and all their files were reviewed by the investigators. Unfortunately, nine of the files reviewed lacked completeness to some extent. All of these, despite scarce data, were still included in our analysis.

The primary outcome of the study was the positivity rate. The relationships between positivity rates, symptoms, clinical suspicion, biopsy delay, biopsy length and corticosteroid treatment were also studied. Patients put on corticosteroid taper courses, regardless of duration, were not included in the “pursuit of treatment” group. Population characteristics as well as complications related to the procedure were also collected.

Data were analyzed using SPSS software (v 24.0) and R (v 3.6.1). Chi2 tests, Fisher’s exact tests and Student’s *t* tests were used for statistical analysis. Test choice was dependent on the characteristics of the variables. *P* values < 0.05 were considered significant. Logistic regressions were also performed.

## Results

The total population was one hundred and twenty-seven (n = 127). Nine patients had an equivocal biopsy result. As such, these patients were included in the demographic and descriptive portion of the analysis, but could not be included for statistical associations and comparisons.

### Population characteristics

The median age of the subjects was 78.0 years old (y-o), with interquartile values of 68 y-o (25) and 83 y-o (75). There was a statistically significant difference in age when comparing patients with positive and negative biopsy results using the Mann–Whitney *U* test (*p* < 0.05). Therefore, older age was associated with an increased likelihood of positive biopsy.

Approximately two-thirds of the patients included in the study (59.7%) were female. Only 5.7% were taking anticoagulation therapy at the time of the biopsy. A total of 48.8% were on antiplatelet therapy at the time of biopsy.

The subspecialties of the referring doctor varied. Rheumatology (33%), internal medicine (25%), neurology (20%) and ophthalmology (15%) accounted for most of the referrals.

Most of the surgeries were performed by an ear–nose–throat specialist (75%), while plastic surgeons (24.2%) also performed some of the procedures. One operation was performed by a general surgeon in our center. A large number of the surgeries were performed in a clinical setting (74.2%), while the remaining procedures took place in an operating room (25.8%). All surgeries were performed under local anesthesia.

The main operator’s training varied. Of all the biopsies, 27.6% were completed by an attending. Others were performed by supervised surgical residents. 44.7% were PGY2.

Table 1 outlines symptoms experienced by patients referred for temporal artery biopsy. A significant association between jaw claudication and specimen positivity (OR 8.1, *p* < 0.05) was found. No other association between symptoms or signs and positivity was established. Additionally, there was no association between a greater number of fulfilled ACR diagnostic criteria and a positive biopsy result (*p* = 0.4).

**Table 1:** Presence of sign/symptom and association with a positive biopsy result

Symptom	Present	Positive biopsy	*p* Value
Headache	87/117 (74.4%)	19/87 (21.8%)	*p* = 0.46
Fever	11/116 (9.5%)	2/11 (18.2%)	*p* = 1.00
Claudication	34/116 (29.3%)	18/34 (52.9%)	*p* < 0.05
Diplopia	13/116 (11.2%)	4/13 (30.8%)	*p* = 0.51
↓ Vision	41/116 (35.3%)	13/41 (31.7%)	*p* = 0.18
↑ ESR/CRP^o^	104/113 (92.0%)	26/104 (25.0%)	*p* = 0.12

^o^Sedimentation rate and c−reactive protein based on upper limits in our center

Association with positive biopsy result, Chi^2^ test or Fisher’s exact tests

In the physical exam, most patients (61.2%) did not show any specific signs. Of these patients, 17.4% and 19.8% showed a diminished pulse in the superficial temporal artery and pain on palpation, respectively.

### Yields of temporal artery biopsy

A positivity rate of 23.7% (CI 95% 16.6–32.6%) was obtained.

### Delays

During data collection, we noticed 2 major factors that seemed to lengthen delays. These were the lack of phone calls for consultation by the referring doctor and preoperative anticoagulant cessation. In our study, longer delays did not have any impact on biopsy positivity (Tables 2, 3).

**Table 2:** Timeline (in days)

	Mean	SD^o^	Median
Consultation—date of biopsy	4.2	5.4	3.0
Date of biopsy—start of treatment	− 4.6	8.0	− 3.0

^o^Standard deviation

**Table 3:** Timelines for positive and negative biopsies (in days)

Biopsy	Biopsy delay	Initiation of treatment
Positive	5.14	− 5.50
Negative	3.88	− 4.29
*p* Value	*p* = 0.30^o^	*p* = 0.52^o^

^o^*p* Value, Student’s *t* tests

### Biopsy characteristics

The mean length of biopsy was 2.48 cm (cm) (SD = 0.89). The median length was 2.35 cm. The minimum and maximum lengths were 0.4 cm and 5.5 cm, respectively. Biopsy length was not associated with a positive biopsy result in the present study. Fifty-three right temporal arteries and 71 left temporal arteries were biopsies. Most of the time, side selection was based on symptoms (53.7%). Other factors that influenced side selection included physical exam (8.9%), imaging (16.3%) or a combination of these factors. A total of 18.7% of the selections were purely random.

### Initial clinical suspicion

**Fig. 1:**
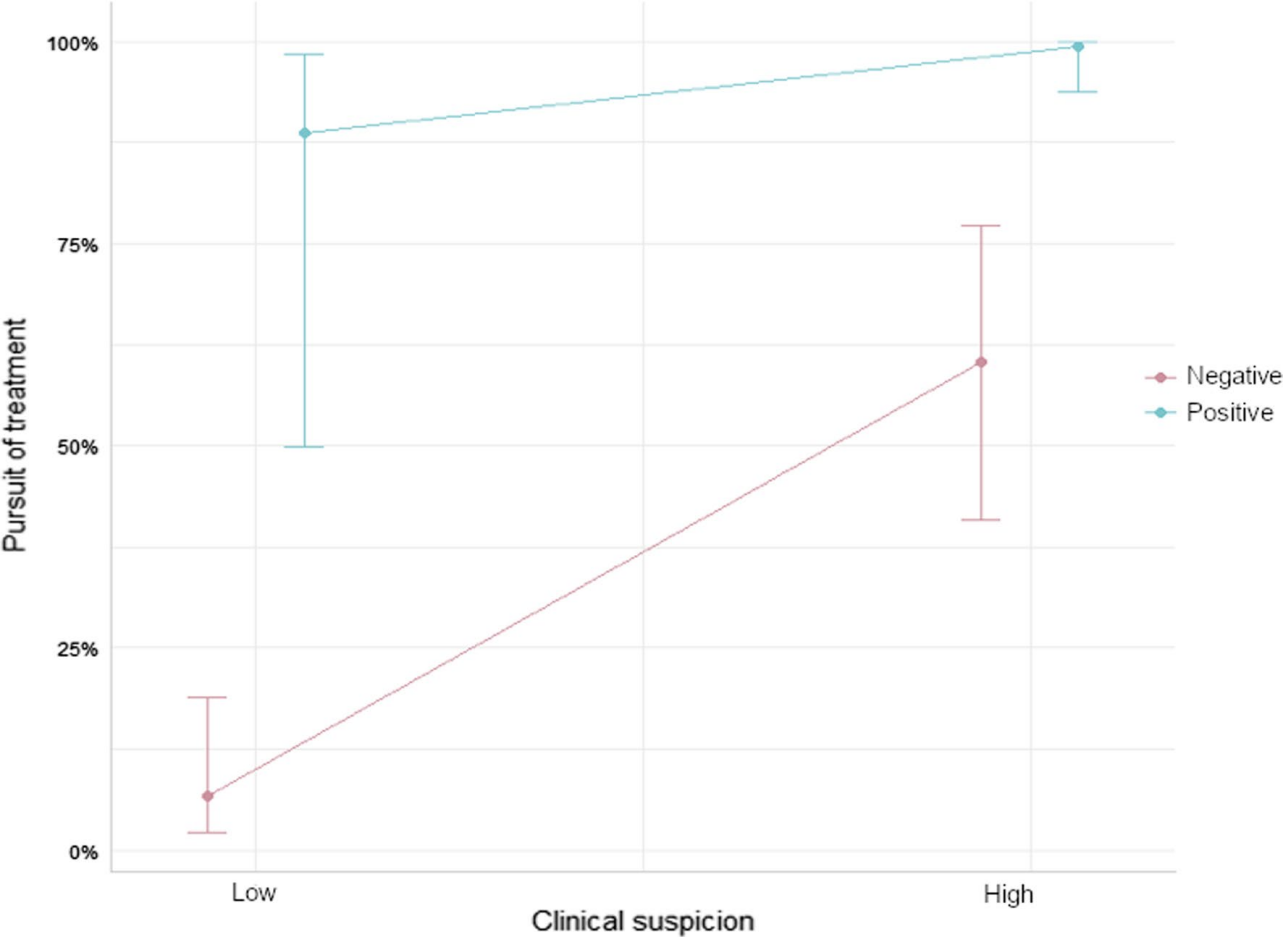
Linear regression for pursuit of treatment and clinical suspicion for positive and negative biopsy results

Initial clinical suspicion was classified as either high or low based on the initial conclusions of the referring physician prior to biopsy. All cases where temporal arteritis was the “most probable” diagnosis were considered high. The ACR diagnostic criteria were not utilized for classification as it was not cited by any of the referring physicians. Overall, fifty cases had a high degree of suspicion, and 71 had a low degree. For 13 patients, this information was not available. Our study showed a significant association between high initial clinical suspicion of disease and specimen positivity (*p* < 0.05). Interestingly, high initial clinical suspicion of disease also correlated with pursuit of treatment following biopsy results, regardless of positivity (*p* < 0.05). This is illustrated in the following linear regression (Fig. 1). Since we used linear regression models, our hypothesis was verified. Finally, the duration of corticosteroid treatment prior to biopsy was not associated with a change in yield.

### Treatment

Among the treated patients, corticosteroids were always the main therapy. Most patients included in the study were treated (83.5%). Treatment was started before the biopsy result in 96.3% of patients who ended up having a positive biopsy result. For 96.2% of patients with a positive biopsy, treatment was continued. Regardless of positivity, treatment was pursued in approximately half of all patients for whom treatment was initiated (48.5%). Of these, 19 patients had a negative biopsy result.

### Complications

There were no immediate complications reported. One case of delayed bleeding occurred. The patient was treated in the emergency room and was sent back home the same day.

## Discussion

Clinical symptoms vary widely when comparing patients with temporal arteritis. Reports also differ in terms of the association of a particular symptom with a positive biopsy. A few studies have reported a link between jaw claudication and giant cell arteritis, such as one study from the Mayo Clinic*,* which showed a 78% positive predictive value [12]. In line with this association, an Odds Ratio of 8.1 for a positive biopsy result in the presence of jaw claudication was found in our study. Our opinion is that clinicians should have a high index of suspicion for patients presenting with jaw claudication. It should also be kept in mind that temporal arteritis can have many different presentations.

The effect of steroid treatment on the biopsy positivity rate is a subject of debate. A cohort study of 535 patients from the Mayo Clinic did not demonstrate any difference between the positivity rates of patients treated with steroids for 14 days or more versus those treated for less than 14 days at the time of biopsy [13]. Other studies have shown a negative impact of prednisone treatment. The positive rate ranged from 78% (< 2 weeks treatment) to 40% (> 4 weeks treatment) in another study [14]. In our study, there was no association between the use of steroid treatment and specimen positivity. This can potentially be attributed to the short delay between referral and biopsy. Almost all of the biopsies were performed in less than 2 weeks. Indeed, most were sent for identification in less than a week, as shown by the mean delay of 4.2 days. This is also highlighted by the fact that in general, steroid treatment was only initiated 4.6 days before biopsy.

A few studies have demonstrated that longer specimens are associated with higher rates of positivity [15]. However, the ideal tissue biopsy length remains unknown.

Our study did not show any significant results with regard to total length. A recent study concluded that length “is not associated with the temporal artery biopsy yield in patients with clinical suspicion of giant cell arteritis” [16]. However, an older retrospective study stated that the positivity rate was significantly higher when the size of the biopsy exceeded 0.7 cm [15]. Another study showed the mean length of biopsy to be 1.84 cm for positive biopsies and 1.29 cm for negative biopsies [17]. Surgeons should consider an average loss of 2.4 mm with specimen fixation [18].

The positivity rate of temporal artery biopsy at our institution is 23.7% (16.6–32.6%). A recent review pooling results from different studies revealed a median yield of 25% for temporal artery biopsy [9]. The interquartile range was 17–33%, suggesting that centers with yields under 17% are overperforming temporal artery biopsies, while those over the 33% limit may be underperforming the procedure.

Only one patient needed a second biopsy for a suspicion of relapse. It has been reported that bilateral biopsies increase the sensitivity of the procedure by 5% [19]. In our center, no bilateral biopsies were reported.

Treatment of patients with a negative temporal artery biopsy was associated with maintenance of corticosteroid treatment when the initial clinical suspicion of arteritis was high. Therefore, temporal artery biopsy may not be necessary for patients with a high initial clinical suspicion of giant cell arteritis.

We initially believed that the diagnostic criteria from the American Rheumatology Association, were a good outline for clinical practice. This has already been advocated in other studies, such as “The Role of Temporal Artery Biopsies in Giant Cell Arteritis”, by Davies and May [8]. In another study, the same group also noted a two-thirds reduction in the number of biopsies when using these criteria [20]. On the other hand, a recent systematic review (2019) performed a meta-regression and reported that the Rheumatology Association criteria did not improve the yield of biopsy. This study also states that these criteria were not intended for diagnostic use and that they are probably not accurate for patients with an ophthalmic subset of temporal artery vasculitis [9]. Our findings are in concordance with the previous study. This emphasizes the diagnosis of temporal arteritis as a clinical diagnosis. Moreover, the results of our study show that doctors are good at suspecting the disease without the need for a biopsy. However, there is a benefit of biopsy for patients for whom the diagnosis is uncertain. As shown in this study, when the initial clinical suspicion is high, regardless of the number of criteria and the results of the biopsy, treatment is typically pursued. This is highlighted by the fact that biopsy results only affect management in approximately 15% of patients [21]. Therefore, our opinion is that temporal artery biopsy should only be used in specific cases. Similar findings have also been published recently (2019) in the plastic surgery literature [22].

The use of Doppler examination represents an ongoing area of research, and its use is becoming more popular. Some studies have reported similar sensitivity results when comparing ultrasound to biopsy [23]. Initial studies led towards the use of ultrasound as a screening method before performing biopsy. Notable disadvantages are that the exam is operator dependent. In our study, the use of Doppler ultrasound was documented but remained scarce. This underlines the fact that ultrasound is not yet widespread in modern medical practice. In our study, both ultrasound and biopsy were ordered simultaneously when ultrasound use was reported. We believe the choice of patients requiring ultrasound should be based on the same clinical grounds as with biopsy. MR studies for temporal arteritis have also been suggested as an alternative to temporal artery biopsy. We believe MRI access and costs might limit the use of this modality in the diagnosis and treatment of giant cell arteritis [24].

Although no major complications were reported, the risks and benefits of the procedure must be balanced when considering surgery. Complications, including the risk of facial nerve injury, can be devastating for a patient. To minimize this risk, dissection should be undertaken over the parietal extension of the temporal superficial artery, which is more posterior [25]. Other complications include bleeding and infection. That said, the risk of complications is low, but surgery should only be used for patients with diagnostic uncertainty. In our study, one case of delayed bleeding occurred. However, the number of minor complications reported in our study might have been underestimated as a result of patients not reporting directly to our establishment. For example, a patient could have consulted his primary care physician outside of the hospital setting for a minor infection. Other added benefits of limiting the number of procedures include decreased costs and improved resource allocation.

Lastly, the risks of long-term corticosteroid therapy are important to consider in the management of these patients. Major risks such as cardiovascular complications, diabetes mellitus, avascular necrosis and osteoporosis are well established [26]. Consequently, we advocate in favor of the procedure if it may alter the long-term treatment of a patient. In our opinion, this is also part of optimizing the use of temporal artery biopsy.

## Limits of the study

This study was an observational retrospective study. This comes with inherent weaknesses such as the inability to form conclusions on the causal relationships observed in this study. This highlights the need for prospective randomized studies in this field, where much uncertainty still prevails. Power might also have been an issue for certain variables studied in the present paper. Finally, since pathology reports were used to establish the patient population, we can hypothesize that some patients treated for giant cell arteritis did not undergo temporal artery biopsy and thus, were not included in the study. Therefore, we can postulate that the total study population could have been greater if not for the retrospective nature of this study.

## Conclusions

The positivity rate of temporal artery biopsy in our study was 23.7%, which is similar to the yields observed in previous studies. There remains debate on the appropriate use of temporal artery biopsy in the diagnosis of giant cell arteritis throughout the literature. Most recent studies state that we are probably overusing temporal artery biopsies [27, 28], but a recent systematic review reported marked heterogenicity in data and still advocated confirmatory biopsy in all cases [9]. This calls for more research in this field, as well as the eventual establishment of new clinical criteria.

Despite all of this, we showed that maintenance of corticosteroid treatment seemed to be the rule, rather than the exception, in patients with a negative biopsy when the initial clinical suspicion of temporal arteritis was high. Therefore, temporal artery biopsy may not be necessary in patients with a high initial clinical suspicion of giant cell arteritis considering that treatment is pursued regardless of biopsy result.

## Data Availability

The datasets generated and analyzed during the current study are available from the corresponding author on reasonable request.

## Abbreviation

Y-o: Years old
